# Impact of Conservative Policies on Resident Physician Recruitment to Southern States

**DOI:** 10.7759/cureus.95341

**Published:** 2025-10-24

**Authors:** Daniel Wells, Anoop Agrawal, Benjamin Doolittle

**Affiliations:** 1 Internal Medicine-Pediatrics, University of Tennessee Health Science Center College of Medicine, Memphis, USA; 2 Internal Medicine, Baylor College of Medicine, Houston, USA; 3 Internal Medicine-Pediatrics, Yale School of Medicine, New Haven, USA

**Keywords:** general internal medicine, med-peds, pediatrics, residency recruitment, survey analysis

## Abstract

Conservative policy shifts in Southern states may be impacting how medical trainees perceive and evaluate residency programs in the region. These states already face challenges with poor health outcomes and physician shortages, and increasingly restrictive laws risk further deterring applicants from prioritizing Southern residency programs. Recent trends in residency recruitment raise urgent concerns about the physician workforce pipeline and warrant deeper study and continued public discussion.

## Editorial

A deepening national political divide has led to increasingly restrictive policies in Southern states. Many Southern legislatures have passed laws restricting abortion, lesbian, gay, bisexual, transgender, and queer (LGBTQ) rights, and diversity, equity, and inclusion (DEI) initiatives. After the Supreme Court overturned *Roe v. Wade* in 2022, multiple near-total abortion bans were enacted across the South [1]. In 2024, at least 523 anti-LGBTQ bills have been proposed in state legislatures [2], mostly from Southern states. Five Southern states (Alabama, Florida, North Carolina, Tennessee, and Texas) have introduced legislation to limit DEI efforts [3].

These laws are influencing residency applications. Data from the Association of American Medical Colleges show a growing gap in US MD seniors applying to residencies in states with abortion bans. States with bans saw a 4.2% decrease across all specialties while those with legalized abortion saw only a 0.6% reduction [4]. In 2021 and 2022, there was no significant difference in application numbers between states [4]. OB/Gyn programs have subsequently seen reductions in states with bans. The 8% drop in internal medicine applications compared to 0.3% the year before, and a 17.3% drop [5] in pediatrics applications - double that of states with legal abortion - are clear warning signs that these policies may be affecting a broader range of applicants.

What is the impact on the match? As members of the Medicine-Pediatrics Program Directors Association, we survey our program directors annually and include questions related to the most recent match. In 2024, nearly 35% of programs in Southern states reported their match was “less or much less favorable,” which was statistically different from other regions. This rose from 2023, when only 22% reported less favorable outcomes. The pediatrics residency match was the lowest in recent years. Of the 249 unfilled spots nationally, 52 (22%) came from Florida, Tennessee, and Georgia - states with recent conservative policy rollouts [5]. To our knowledge, data for internal medicine programs remain unknown.

Why does this matter? While we cannot prove causation, conservative policies may deter applicants from ranking Southern programs highly despite their academic quality. Anecdotally, program directors from Southern programs report that these policies deter many applicants. Though all Southern med-peds programs filled in the match, these trends risk leaving programs unfilled or unable to attract top candidates. Building a pipeline for medical trainees in Southern states is crucial. As defined by the US Census Bureau, seven out of the 10 states with the worst health outcomes are in the South. Furthermore, 15 of 16 states in this region rank in the bottom 50% for health outcomes [6]. The Southern United States urgently needs high-quality physicians but faces a shrinking pipeline. In 2019, before these conservative policies, 52%-59% of graduates from Southern states left the region for residency [7]. With new social policies, we are concerned that this outmigration will accelerate, worsening health outcomes.

What should we do? This issue needs further study. Our survey may be among the first to explore program directors’ perceptions by region using both quantitative and qualitative insights into how politics might affect rank lists. While many factors influence rankings - virtual interviews, second looks, etc. - these changes affect all programs equally. Although “less or much less favorable” is subjective, program directors closely track match outcomes. We believe our survey is a canary in the Sun Belt, signaling a broader trend. This issue deserves public discussion before its impact takes a toll.
